# Effect of environment and genotypes on the physicochemical quality of the grains of newly developed wheat inbred lines

**DOI:** 10.1002/fsn3.313

**Published:** 2015-11-17

**Authors:** Noha I. A. Mutwali, Abdelmoniem I. Mustafa, Yasir S. A. Gorafi, Isam A. Mohamed Ahmed

**Affiliations:** ^1^ Department of Food Science and TechnologyFaculty of AgricultureUniversity of KhartoumShambat13314KhartoumSudan; ^2^ Wheat Research ProgramAgricultural Research CorporationP.O. Box: 26Wad MedaniSudan

**Keywords:** Falling number, gluten quality, growing environment, wheat genotypes

## Abstract

To meet the increased demand for wheat consumption, wheat cultivation in Sudan expanded southward to latitudes lower than 15°N, entering a new and warmer environment. Consequently, wheat breeders developed several wheat genotypes with high yields under these environmental conditions; however, the evaluation of the end‐use quality of these genotypes is scarce. In this study, we assessed the end‐use quality attributes of 20 wheat genotypes grown in three different environments in the Sudan (Wad Medani, Hudeiba, and Dongola). The results showed significant differences (*P *≤* *0.01) in all quality tests among environments, genotypes and genotypes Versus environments. The findings obtained, covered wide ranges of test weight (TW, 76.6–85.25 kg/hL), thousand kernel weight (TKW, 28.70–48.48 g), protein (PC, 9.96–14.06%), wet gluten (WG, 28.63–46.53%), gluten index (GI, 36.36–92.77%), water holding capacity (WHC, 168.42–219.32%), falling number (FN, 508.00–974.67 sec), and sedimentation value (SV, 19.00–40.00 mL). Analysis of the traits, genotypes, and traits versus genotypes showed varied correlations in the three growing environments. The genotype G3 grown in either one or all of the three environments exhibits worthy performance and stability for most of the tested quality traits. The crossing of this genotype with high yield genotypes could produce cultivars with sufficient quality and marketability.

## Introduction

Wheat is an important and most widely cultivated food crop in the world. This crop played a central role in combating hunger and improving the global food security. Wheat is ranked second in total cereal production behind corn, with rice being the third (FAO, 2012). The grains of this plant provide about 20% of all calories and proteins consumed by people on the globe (Shiferaw et al. 2013). In recent years, demand for wheat has significantly increased as a result of the global population growth, and thus wheat production has a strategic role in food security and the world economy. As a result, horizontal expansion of wheat production has arisen in recent years by moving wheat into nontraditional areas formerly considered unacceptable for production. However, the global warming introduced various abiotic stresses such as drought, temperature extremes, and salinity that adversely affect the yield and grains quality of wheat (Huseynova and Rustamova 2010). To meet the demands of future population's explosions and ensure grain production in these environments, cultivars must be developed and evaluated for their high yield and high quality. Thus, the objective of wheat breeders is to produce well‐adapted and high‐yielding varieties with finest end‐use quality (Lopes et al. 2012; Li et al. 2013).

In Sudan, wheat is the second most essential cereal food and the main staple food for many peoples in both rural and urban areas. This crop is traditionally cultivated in the northern region of Sudan where the winter conditions are favorable for plant growth and grain yield. However, in the last decades wheat cultivation in Sudan expanded southward to latitudes lower than 15°N, entering a new and warmer environment and inhabiting most of the irrigated sectors in central and northern states (Elsheikh et al. 2015). The average annual production of wheat during the period 2009–2013 was 242,000 tons and is forecasted to rise to 320,000 tons in 2014 with 32% change (FAO, 2014). Nevertheless, the rate of wheat grain production in the Sudan is far below the consumption needs. High temperature and drought stresses, low nitrogen content, and lack of quality seeds of improved varieties are the main constraints limiting wheat production in Sudan (Ali et al. 2006; El Siddig et al. 2013). To overcome these limitations wheat breeders have developed several varieties and inbred lines with enhanced tolerance to most of these stresses (Elahmadi 1996; Ali et al. 2006), and with better grain yield and quality. Although the grain yield of these advanced wheat lines has been extensively studied by many researchers, reports on the end‐use quality of these lines are rare (Ali et al. 2006). Therefore, the primary objective of this study was to examine the effect of growing environment on end‐use quality characteristics of twenty wheat genotypes grown in three different environments (Wad Medani, Hudeiba, and Dongola) in the Sudan.

## Materials and Methods

### Plant materials and field trials

In this study, 20 wheat genotypes representing a broad range of yield and adaptability to the environment of the Sudan were used (Table 1). These genotypes were developed through extensive wheat breeding programs at the Agricultural Research Corporation (ARC), Gazira, Sudan. All materials were grown for two constitutive seasons (2003/2004 and 2004/2005) in three different environments (Dongola, Hudeiba, and Wad Medani) representing both the traditional and new wheat growing environments in the Sudan. The three growing environments are characterized by their different soil and environmental conditions with no precipitation during the whole crop cycle. Dongola is located in Northern State (Lat. 19° 08′ N, Long. 30° 40′ E, and Alt. 240 m) with a warm (average temperature 21.8°C) and dry winter. The soil of Dongola is classified as sandy clay loam with very low organic matters (>5%), high water permeability, and a pH of 8.0. Whereas, Hudeiba is located in the Nile State (Lat. 17° 34′ N, Long. 33° 56′ E, and Alt. 350 m) and has warm (average temperature 24.2°C) and dry winter. The soil of Hudeiba is classified as karusoil clay (contained 4% sand, 40% silt, and 56% clay) with little nitrogen (360 ppm), phosphorus (8 ppm), and organic carbon, and a pH of 8.1. While Wad Medani is located in central Sudan (Lat. 14° 24′ N, Long. 29° 33′ E, and Alt. 407 m) having a slightly hot (average temperature 26.8°C) and dry winter. The soil type of Wad Medani is heavy cracking vertisoil (58–66% clay) with very low water permeability, organic carbon (0.35%), nitrogen (0.03%), phosphorus (4 ppm), and a pH 8.3

**Table 1:** Genotypes used in the current study

Genotypes code	Pedigree/Variety
G1	ELNeilain
G2	Debeira
G3	RGO/SERI/TRAP//Bow
G4	KAU2 * CHEN//BCN. CMB
G5	SON64/SRC – LR64A) G155
G6	427F4/2000‐1
G7	PYT#23 (DWR39xCONDOR “S”)14PxT
G8	KAUZ “S” 6 57C1‐3‐6.2‐2‐1‐2
G9	TEVEE “S”/SHUHA “S”
G10	N5732/HER//CASKOR
G11	ELNEILAIN/SASARIBE
G12	CONDOR “S”/14PYT//DWR39
G13	VERONA/KAUZ//KAUZ
G14	ELNEILAIN/DEBEIRA
G15	OASIS/KUAZ//3 * BCN
G16	CONDOR “S”/BALADI//DEBEIRA
G17	DH5
G18	DH8
G19	IHSGE # 19
G20	IHSGE # 20

In the three growing environments, the experiments were structured in a randomized complete block design with three replications. After soil harrowing and leveling, the seeds were seeded manually in rows of 0.2 m apart in plots consisting of 4 rows of 5 m length at a seeding rate of 120 kg/ha. The seeds were treated with Gaucho (Imidacloprid 35% WP) at a rate of about 1 g/1 kg seed to control pests mainly termites and aphids. Phosphorus was applied by furrow placement prior to sowing at the rate of 43 kg P_2_O_5_/ha, while nitrogen, in the form of urea, was implemented before the second irrigation at a rate of 86 kg N/ha. Irrigation intervals were every 10–12 days, and weeding was carried out manually at least twice.

### Samples preparation

The wheat grains were manually cleaned and then the grains thousand kernels and test weights were evaluated. The samples were tempered and milled into straight grade flour (72% extraction rate) using a Brabender Quadrumat Junior Mill (Brabender, GmbH & Co. KG, Duisburg, Germany). After that, the flour samples were placed in a separate plastic container and stored in a deep freezer until used for biochemical analysis. Three independent replicates of each sample were used for biochemical analysis.

### Chemical composition

Moisture, ash, crude protein (N × 5.7), and fat content of the flour samples were measured according to the official standard method (AACC, 2000).

### Gluten quantity and quality

Wet gluten (WG), dry gluten (DG), water holding capacity (WHC), and gluten index (GI) were determined according to the standard method 38–12.02 (AACC, 2000) using a Glutomatic 2200 systems and a Perten 2015 centrifuge (Perten Instrument, AB, Huddinge, Sweden).

### Falling number

Falling number (FN) of wheat flours was determined using the falling number instruments following the Official Method 56–81.03 (AACC, 2000) and expressed on 14% moisture basis.

### Sedimentation value

Zeleny sedimentation value of wheat flours was measured according to the standard method 56–60.01 (AACC, 2000) and expressed on 14% moisture basis.

### Statistical analyses

For the grains of individual wheat genotypes grown in each environment, the data of three independent experiments were first separately analyzed, and then the results were combined to determine the interactive effects of genotypes and growing environments. The data were assessed by analysis of variance (Gomez and Gomez 1984) and Duncan's multiple range test (DMRT). Correlation coefficients among all quality traits were evaluated based on the means of all genotypes in the individual environment using Stat View software. Exploratory multivariate statistical analysis of the data was performed using HJ‐biplot methods included in the MULTBIPLOT software (Vicente‐Villardón 2010). The HJ‐Biplot method allows the plotting of both genotypes and wheat quality traits with an optimum quality of representation and hence provides easy and fast information about the interrelation of the plotted data. To ascribe a set of individuals to a particular group, we performed hierarchical clustering analysis with the Euclidean distance using the principal components scores and the Ward's technique as the process of linkage. Significance was accepted at *P *≤* *0.05, *P *≤* *0.01, and *P *≤* *0.001.

## Results and Discussion

### Grain physical characteristics

Thousand kernels (TKW) and test weight (TW) of wheat genotypes grown in three different environments are shown in Table 2. The TKW and TW of all genotypes were significantly varied (*P *≤* *0.01) between the three environments. These results revealed that the variation in the environmental and soil conditions between the three environments could contribute to the differences in the wheat grain weight. In addition, the interaction between the wheat genotypes and the growing environment was also significant (*P *≤* *0.01) for both traits. The highest mean values of TKW and TW were observed for G5 at Hudeiba and G19 at Dongola while the lowest values were obtained for G12 at Wad Medani and G3 at Hudeiba, respectively. Regarding the environments, the highest mean values of TKW and TW for all genotypes were at Hudeiba and Dongola while the lowest values were at Wad Medani and Hudeiba, respectively. Throughout all the three environments, the mean TW was in the range of 78.02–82.83 kg/hL, which indicates that all wheat genotypes exhibit well‐filled grains (Kaya and Akcura, 2014; Li et al. 2013). Overall, the results of the TKW and TW demonstrated that the environmental conditions (temperature and soil fertility), agronomic practices (irrigation and fertilization), and wheat genotypes could affect the grain physical characteristic and hence the flour yield and end‐use quality. Previous reports showed that environmental conditions and fertilizers application had a significant impact on the TKW and TW of various wheat genotypes (Lopes et al. 2012; Mohammadi 2012; Li et al. 2013; Bouacha et al. 2014; Kaya and Akcura 2014). In addition, water deficit and elevated temperatures above average during grain filling reported to reduce the TKW for winter wheat (Erekul and Kohn 2006). Mohammadi (2012) concluded that wheat cultivars capable of maintaining high TKW under heat stress appeared to possess a greater tolerance for warm environments. While Lopes et al. (2012) suggested the use of TKW for selection of wheat genotypes under a warm environment of the Sudan.

**Table 2:** Thousand kernel weight (g) and test weight (kg/ha) of 20 wheat genotypes grown in three environments

Genotypes	1000 Kernel weight (TKW)				Test weight (TW)			
	Wad Medani	Hudeiba	Dongola	Mean	Wad Medani	Hudeiba	Dongola	Mean
G1	35.81^tuv^	46.21^a^	36.97^ij^	39.66^fg^	82.03^i^	80.40^nop^	84.54^b^	82.32^b^
G2	35.20^v^	44.82^cd^	36.76^rs^	38.92^hi^	82.53^fg^	79.65^rs^	82.45^gh^	81.54^f^
G3	36.38^st^	44.24^de^	40.44^hi^	40.35^de^	77.33^v^	76.60^w^	80.14^opq^	78.02^m^
G4	35.52^uv^	44.53^d^	41.08^gh^	40.37^d^	81.45^jk^	80.89^lm^	84.40^b^	82.24^b^
G5	39.48^jkl^	48.48^a^	43.66^ef^	43.87^a^	82.50^g^	80.89^lm^	83.58^cd^	82.32^b^
G6	37.17^pqr^	45.50^bc^	38.98^klm^	40.55^cd^	81.43^k^	79.63^rs^	83.45^d^	81.50^fg^
G7	35.86^tuv^	39.70^ijk^	36.18^stu^	37.25^j^	82.11^hi^	79.95^qr^	79.55^st^	80.54^i^
G8	31.22^y^	36.54^rst^	33.52^xy^	33.76^l^	82.08^hi^	78.11^u^	82.45^gh^	80.88^h^
G9	33.77^xy^	43.81^de^	39.71^ijk^	39.09^h^	81.55^jk^	80.25^op^	83.35^d^	81.71^ef^
G10	33.63^xy^	44.76^c^	39.38^ijk^	39.25^gh^	79.21^t^	77.50^v^	80.50^no^	79.07^l^
G11	37.88^nop^	44.32^de^	40.83^gh^	41.01^bc^	82.70^efg^	81.50^jk^	84.30^b^	82.83^a^
G12	28.70^z^	40.75^h^	34.30^wx^	34.58^K^	80.25^op^	80.05^pq^	84.35^b^	81.55^f^
G13	37.84^opq^	44.64^cd^	41.73^g^	41.40^B^	81.81^ij^	79.54^st^	82.66^fg^	81.34^g^
G14	31.42^y^	37.63^opq^	34.70^w^	34.58^K^	78.11^u^	81.40^k^	79.55^st^	79.68^j^
G15	30.09^z^	38.88^klm^	35.26^v^	34.74^k^	81.45^jk^	80.25^op^	83.85^c^	81.85^de^
G16	30.71^z^	35.52^uv^	33.17^yz^	33.13^m^	82.01^i^	81.30^k^	83.00^e^	82.10^bc^
G17	31.24^y^	46.14^b^	38.22^mno^	38.54^i^	82.75^efg^	81.20^kl^	82.90^ef^	82.28^b^
G18	33.42^y^	42.82^f^	39.20^jkl^	38.48^i^	79.31^st^	79.14^t^	79.55^st^	79.33^k^
G19	31.91^y^	41.55^g^	38.72^lmn^	37.39^j^	80.73^mn^	79.95^qr^	85.25^a^	81.97^cd^
G20	35.14^v^	44.71^cd^	39.96^ij^	39.94^ef^	80.50^no^	79.55^st^	82.45^gh^	80.83^h^
Mean	34.12^c^	42.78^a^	38.14^b^	38.34	81.09^b^	79.88^c^	82.61^a^	81.19

Means followed by the same letter are not significantly different (*P *≤* *0.01) from each other, according to Duncan's multiple range test.

The grain physical characteristics TKW and TW have no correlation with all other quality traits of all genotypes grown in Wad Medani, whereas they showed some correlations with the quality traits in other environments (Table 3). At Hudeiba, TKW showed a significant positive correlation (*P <* *0.05, *r* = 0.49) with PC, while it showed highly significant negative correlation (**P <* *0.01, *r* = −0.61) with GI. The TW, on the other hand, showed significantly negative (*P <* *0.05) correlations with the SV at Hudeiba (*r* = −0.44) and with GI at Dongola (*r* = −0.50). These results suggest that the association between grain physical characteristics (TKW and TW) and the flour quality traits mainly PC, GI, and SV depend primarily on the environmental conditions rather than the genetic makeup of the cultivar. Similarly, positive correlations between TKW and PC have been reported for wheat cultivars and landraces grown in the subhumid region following K‐fertilizers treatment (Bouacha et al. 2014). By contrast, negative correlations between grain physical characteristics and flour PC and SV for other wheat genotypes have also been reported (Ozturk and Aydin 2004; Tahir et al. 2006).

**Table 3:** Correlation coefficient between the physicochemical quality parameters of wheat genotypes grown in three different environments (Wad Medani, Hudeiba, and Dongola)

	TKW	AC	MC	TW	GI	WHC	FN	SV	WG	DG	PC
Wad Medani											
AC	0.10										
MC	0.38	0.03									
TW	0.19	−0.17	0.21								
GI	−0.04	0.19	−0.34	−0.04							
WHC	0.18	−0.32	−0.20	−0.25	**0.53** *						
FN	0.17	0.25	0.19	−0.32	0.37	0.35					
SV	−0.16	−**0.53** *	0.06	−0.12	−0.25	0.11	−0.23				
WG	−0.21	−0.37	0.22	−0.06	−**0.83** ***	−0.44	−0.39	**0.55** *			
DG	−0.21	−0.22	0.24	0.08	−**0.86** ***	−**0.68** ***	−**0.45** *	0.42	**0.94** ***		
PC	−0.19	−0.37	0.21	−0.20	−0.43	0.02	0.03	**0.69** ***	**0.64** ***	**0.50** *	
FC	−0.36	0.19	−0.26	−0.43	0.25	0.11	−0.08	0.35	0.04	−0.02	0.07
Hudeiba											
AC	−0.27										
MC	0.01	−0.17									
TW	−0.05	−**0.47** *	0.29								
GI	−**0.61** **	−0.20	−0.02	0.02							
WHC	−0.22	−0.06	0.24	0.10	0.17						
FN	−0.02	0.07	−0.14	−0.03	−0.01	−0.05					
SV	−0.15	−0.15	−0.01	−**0.44** *	0.25	0.30	**0.47** *				
WG	0.41	0.02	0.02	−0.36	−**0.47** *	−0.39	**0.48** *	**0.45** *			
DG	0.39	0.04	−0.03	−0.35	−0.42	−**0.64** ***	0.41	0.29	**0.96** ***		
PC	**0.49** *	−0.02	−0.18	−0.32	−**0.61** **	−0.40	0.36	0.35	**0.83** ***	**0.80** ***	
FC	−0.20	0.09	−0.31	−0.17	0.24	0.10	**0.67** ***	**0.59** **	0.26	0.21	0.26
Dongola											
AC	−0.05										
MC	0.15	−0.24									
TW	0.10	−0.02	−0.18								
GI	−0.41	0.02	−0.26	−**0.50** *							
WHC	0.42	−0.07	−0.20	−0.28	0.25						
FN	−0.39	0.21	−**0.67** ***	−0.02	0.42	0.04					
SV	−0.17	−**0.51** *	0.25	−0.44	**0.50** *	0.15	−0.11				
WG	0.05	−**0.49** *	0.33	0.20	−**0.48** *	−0.28	−0.35	0.21			
DG	−0.09	−0.31	0.35	0.26	−**0.51** *	−**0.70** ***	−0.31	0.05	**0.87** ***		
PC	0.12	−0.10	**0.54** *	−0.11	−0.03	−0.16	−0.22	0.31	**0.52** *	**0.48** *	
FC	0.02	0.19	0.21	−0.14	−0.18	−0.17	0.07	−0.14	−0.16	0.02	0.14

TKW, Thousand‐kernel weight; AC, Ash content; MC, Moisture content; TW, test weight; GI, Gluten index; WHC, Water holding capacity; FN, Falling number; SV, Sedimentation value; WG, Wet gluten; DG, Dry gluten; PC, Protein content; FC, Fat content.

Values in bold are significant at *P <* *0.05; **P <* *0.01; ***P <* *0.001.

### Chemical composition

The results of moisture, ash, protein, and fat content are presented in Table 4. The moisture content (MC) was in the range of 11.38–13.13%, 10.85–12.48%, and 10.21–12.21% for the genotypes grown at Wad Medani, Hudeiba, and Dongola, respectively. Significant differences (*P *≤* *0.01) in MC among all environments, indicating that environments had influenced the flour moisture content. Although it has no correlation with other traits in Wad Medani and Hudeiba, MC demonstrated extremely negative correlation (***P <* *0.001, *r* = −0.67) with FN and positive correlation (*P <* *0.05, *r* = 0.54) with PC at Dongola (Table 3). The highest mean value for MC (12.31%) among all genotypes was observed in Wad Medani while lowest value (11.61%) was scored in Dongola. Regardless of the environment, the highest MC was recorded for G5 that was significantly different (*P *≤* *0.01) from all other genotypes, while the lowest value was observed for G6. Regarding the interaction between wheat genotype and growing environment, the highest MC (13.13%) was obtained for G13 grown at Wad Medani, whereas the lowest value (10.21%) was noticed for G2 grown at Dongola. The results achieved agreed with values obtained by Makawi et al. (2013) who stated that the moisture content of Sudanese wheat cultivars ranged from 10.40 to 12.07%. The variation in MC of the different wheat genotypes may be due to the variations in environmental conditions between the three environments, the genotypes, and their interaction. Moisture content is mostly affected by relative humidity at harvest and during storage (Makawi et al. 2013).

**Table 4:** Moisture, ash, protein, and fat content (%) of 20 local wheat genotypes grown in three environments

Variety/Lines	Moisture (MC)				Ash (AC)				Protein (PC)				Fat (FC)			
	Wad Medani	Hudeiba	Dongola	Mean	Wad Medani	Hudeiba	Dongola	Mean	Wad Medani	Hudeiba	Dongola	Mean	Wad Medani	Hudeiba	Dongola	Mean
G1	12.79^b^	11.44^z^	11.60^uvwxyz^	11.94^defg^	0.69^efghi^	0.61^jklmno^	0.61^jklmno^	0.64^fgh^	10.58^u^	12.31^lmn^	12.12^mno^	11.67^h^	1.15^lmn^	1.03^pqr^	0.93^r^	1.04^g^
G2	12.63^c^	11.31^z^	10.21^z^	11.38^j^	0.67^fghijk^	0.68^fghij^	0.71^defgh^	0.68^cde^	9.97^w^	13.03^fg^	12.04^no^	11.68^h^	1.44^de^	1.74^a^	1.13^mno^	1.43^a^
G3	12.11^Ijk^	11.71^qrstuvwx^	12.15^hijk^	11.99^cd^	0.73^cdef^	0.76^bcd^	0.73^cdef^	0.74^b^	11.15^q^	13.60^bcd^	12.36^klm^	12.37^c^	1.38^efg^	1.41^def^	1.46^d^	1.41^a^
G4	12.07^jklm^	11.65^rstuvwx^	11.82^nopqr^	11.85^efg^	0.63^ijklmn^	0.57^o^	0.79^b^	0.66^efg^	10.94^qrs^	14.06^a^	13.87^ab^	12.96^b^	0.99^r^	1.35^fgh^	1.22^jkl^	1.19^e^
G5	12.15^hijk^	12.48^cde^	12.18^hij^	12.27^a^	0.61^jklmno^	0.61^klmno^	0.66^ghijk^	0.63^gh^	9.99^w^	13.24^ef^	12.55^jkl^	11.92^g^	1.03^pqr^	1.00^r^	0.93^r^	0.99^h^
G6	12.27^fghi^	11.22^z^	10.27^z^	11.25^k^	0.63^ijklmn^	0.63^ijklm^	0.72^cdefg^	0.66^efg^	10.06^w^	11.05^q^	11.96^o^	11.02^i^	0.96^r^	1.01^qr^	1.17^klmn^	1.04^g^
G7	12.37^efg^	11.60^uvwxyz^	12.06^jklm^	12.01^bc^	0.63^ijklmn^	0.77^bc^	0.59^mno^	0.66^ef^	10.36^v^	12.63^ijk^	13.55^cd^	12.18^def^	1.05^opqr^	1.23^jk^	1.09^nopq^	1.12^f^
G8	12.61^c^	11.08^z^	11.81^nopqrs^	11.83^fg^	0.69^fghi^	0.66^ghijk^	0.66^ghijkl^	0.67^ef^	11.40^p^	12.09^mno^	12.79^ghij^	12.09^ef^	1.01^qr^	1.45^def^	1.40^def^	1.28^d^
G9	12.62^c^	11.52^yz^	12.19^ghij^	12.11^b^	0.47^p^	0.50^p^	0.59^mno^	0.52^j^	13.40^de^	13.13^f^	13.76^b^	13.43^a^	0.91^r^	1.15^lmn^	1.35^fgh^	1.14^f^
G10	12.33^efgh^	11.53^yz^	12.21^ghij^	12.02^bc^	0.59^mno^	0.64^ijklmn^	0.59^mno^	0.61^hi^	10.66^tu^	13.00^fg^	13.42^de^	12.36^c^	1.44^de^	1.14^r^	1.27^hij^	1.28^d^
G11	12.61^c^	11.55^wxyz^	11.85^nopq^	12.00^cd^	0.75^bcde^	0.56^o^	0.71^defgh^	0.67^ef^	11.53^p^	11.53^p^	13.16^f^	12.07^efg^	1.26^ij^	1.11^mnop^	1.23^jk^	1.20^e^
G12	12.24^fghij^	11.81^nopqrs^	11.93^lmno^	11.99^cd^	0.62^jklmno^	0.74^bcde^	0.72^cdefg^	0.70 ^cd^	12.25^lmn^	12.63^ijk^	13.68^bc^	12.85^b^	1.41^def^	1.13^mno^	1.10^mnop^	1.21^e^
G13	13.13^a^	11.18^z^	11.77^opqrstu^	12.02^bc^	0.85^a^	0.78^bc^	0.71^defgh^	0.78^a^	10.77^stu^	13.19^f^	13.03^fg^	12.33^cd^	1.13^mno^	1.29^hij^	1.46^d^	1.29^d^
G14	11.91^mnop^	11.97^jklmn^	11.63^stuvwxy^	11.83^fg^	0.81^a^	0.63^ijklmn^	0.78^bc^	0.74^b^	10.94^qrs^	9.96^w^	13.51^cd^	11.47^h^	1.32^ghi^	1.29^hij^	1.00^r^	1.20^e^
G15	12.56^cd^	10.85^z^	11.65^rstuvwx^	11.68^h^	0.61^jklmno^	0.71^defg^	0.70^defgh^	0.68^def^	11.04^qr^	12.97^fgh^	13.05^fg^	12.35^c^	1.35^fgh^	1.46^d^	1.46^d^	1.42^a^
G16	11.78^opqrst^	12.41^def^	11.57^vwxyz^	11.92^cdef^	0.67^fghij^	0.61^jklmno^	0.71^defgh^	0.66^ef^	9.59^x^	11.19^q^	11.40^p^	10.72^j^	1.23^jk^	1.24^ijk^	1.18^klm^	1.21^e^
G17	11.38^z^	11.47^z^	11.64^rstuvwxy^	11.49^i^	0.70^defgh^	0.49^p^	0.65^hijklm^	0.61^hi^	10.88^st^	13.08^fg^	12.73^hij^	12.23^cde^	1.42^def^	1.54^c^	0.94^r^	1.30^d^
G18	12.22^ghij^	11.61^tuvwxy^	11.54^xyz^	11.79^g^	0.72^cdefg^	0.78^bc^	0.75^bcde^	0.75^b^	11.35^p^	12.39^klm^	12.36^klm^	12.03^fg^	1.64^b^	1.29^hij^	1.23^jk^	1.38^b^
G19	12.10^jkl^	11.74^pqrstu^	11.23^z^	11.69^h^	0.70^efgh^	0.78^bc^	0.67^fghijk^	0.71^bc^	10.11^vw^	11.13^q^	12.15^mno^	11.13^i^	1.64^b^	1.26^ij^	1.05^opqr^	1.32^cd^
G20	12.37^efg^	12.05^jklmn^	11.25^z^	11.89^defg^	0.58^no^	0.59^mno^	0.60^lmno^	0.59^i^	12.81^ghi^	12.55^jkl^	13.11^f^	12.82^b^	1.65^b^	1.40^def^	1.01^qr^	1.35^bc^
Mean	12.31^a^	11.61^b^	11.63^b^	11.85	0.67^b^	0.65^c^	0.68^a^	0.67	10.99^c^	12.44^b^	12.83^a^	12.08	1.27^a^	1.27^a^	1.18^b^	1.24

Means followed by the same letter are not significantly different (*P *≤* *0.01) from each other, according to Duncan's multiple range test.

Statistical analysis showed significant differences (*P *≤* *0.01) in AC among the growing environments, indicating that the environment had affected the flour ash content (Table 4). Throughout the three areas, the highest mean value (0.68%) of AC for all genotypes was obtained at Wad Medani while the lowest value (0.65%) was observed at Hudeiba. The AC showed significantly negative correlations (*P <* *0.05) with SV at Wad Medani (*r* = −0.53) and Dongola (*r* = −0.51), with TW at Wad Medani (*r* = −0.47), and with WG and Dongola (*r* = −0.49) (Table 3). Among wheat genotypes grown in the three environments, G13 had the highest AC, whereas G9 had the lowest. The differences seen in the AC in the present study may be attributed to differences in wheat genotypes and environmental conditions (temperature and soil conditions) as well as fertilizers application (Makawi et al. 2013).

Wheat grain protein is of primary importance in determining the end use quality of the flour and variations in both protein content and composition could significantly affect the flour quality. The crude protein (PC) content was found to be in the range of 9.59–13.40%, 9.96–14.06%, and 11.40–13.87% for the growing environments Wad Medani, Hudeiba, and Dongola, respectively (Table 4). The results revealed significant differences (*P *≤* *0.01) in the PC among the wheat genotypes and their interaction with the growing environments. These findings indicated that both the genotypes and the growing environment had influenced the flour protein content. Throughout the three growing environments, the highest mean value (13.43%) of PC was found for genotypes grown in Dongola while the lowest value (10.99%) was observed in those grown in Wad Medani. Regarding the interaction, the highest value was obtained for G4 in Hudeiba and Dongola while the lowest value was obtained for G16 at Wad Medani. This result agreed with the outcome of Elmobarak et al. (2004) who stated that wheat grown at Wad Medani gave lower grain protein content compared to that of North Sudan. The variation in PC in the current study may be due to variation in environmental conditions such as heat, drought, and soil fertility (Elmobarak et al. 2004), as well as genotypes. Tolbert (2004) found out that increasing nitrogen fertilizer increased the protein content of flour and the arrival time of dough. Many experiments and practical experience of wheat researchers show that the protein content of the grains and flours is greatly depend on agronomical practices, genotypes, soil N content, heat, and drought stresses (Morris et al. 2004; Tahir et al. 2006; Li et al. 2013; Bouacha et al. 2014; Kaya and Akcura 2014). In the current study, PC showed varied degrees of positive correlations with both WG and DG at the three growing environments (Table 3). It showed highly (***P <* *0.001) positive correlation with WG at Wad Medani (*r* = 0.64) and Hudeiba (*r* = 0.83) and with DG at Hudeiba (*r* = 0.80) as well as a positive (*P <* *0.05) correlation with WG at Dongola (*r* = 0.52) and DG at Wad Medani (*r* = 0.50) and Dongola (*r* = 0.48). Although, these results suggest the dependence of these quality traits on the genotypes rather than the growing environment. However, the crop management practices could have some impacts on these characters. Similar to our findings, previous reports showed the definite interrelation between PC, WG, and DG (Ozturk and Aydin 2004; Kaur et al. 2013; Kaya and Akcura 2014).

There were significant differences (*P *≤* *0.01) in fat content (FC) within the three environments and wheat genotypes (Table 4). Regarding the growing area, the highest mean value of FC was obtained for Hudeiba and Dongola while the lowest value was obtained for Wad Medani. Among genotypes, G2 showed that the highest FC while G5 showed the lowest value. Overall all, MC, AC, PC, and FC of the wheat genotypes of the current study depended greatly on the genotypes, the growing environment and the interaction between genotypes and environments.

### Gluten quantity and quality

Mean values of wet gluten (WG) of wheat genotypes grown in the three environments were significantly varied (*P *≤* *0.01) depending on the differences in the genotypes and growing environments as well as the interaction between these factors (Table 5). The mean values of WG ranged from 32.39 to 46.94%, 28.63 to 46.53%, and 35.5 to 44.26% for the wheat genotypes grown at Wad Medani, Hudeiba, and Dongola, respectively. Regardless of the growing environment, the WG contents of all wheat genotypes in the current study are more than 28% and are, therefore, at a high to the very high range. Recently, in a multienvironment trial for Turkish wheat genotypes the wet gluten content was varied from 28 to 37% depending on the variation in the environment, genotype, and their interaction (Kaya and Akcura 2014). The highest mean value for WG was obtained for G12 (46.94%) and G20 (46.53%) grown at Wad Medani and Hudeiba, respectively, while the lowest value was recorded for G14 (28.63%) cultivated at Hudeiba. Throughout the growing environment, both Dongola and Hudeiba are suitable conditions for WG content compared to Wad Medani. This result indicates that the growing environment influence WG content of these genotypes and hence the gluten quality. The variation in WG could be attributed to the differences in the genotypes, agronomical practices, and environmental conditions such as temperature and soil fertility. Similarly, significant variation in WG content due to the difference in wheat genotypes and growing environment has been reported (Kaya and Akcura 2014).

**Table 5:** The values (%) of wet and dry gluten, gluten index, and water holding capacity of twenty local wheat genotypes grown in three environments

Genotypes	Wet gluten (WG)				Dry gluten (DG)				Gluten index (GI)				Water holing capacity (WHC)			
	Wad Medani	Hudeiba	Dongola	Mean	Wad Medani	Hudeiba	Dongola	Mean	Wad Medani	Hudeiba	Dongola	Mean	Wad Medani	Hudeiba	Dongola	Mean
G1	37.32^klmn^	38.78^hijk^	38.43^hijk^	38.18^fg^	12.67^efghi^	13.58^cdefg^	12.60^fghi^	12.95^fgh^	64.04^lmno^	50.42^qr^	69.22^ijkl^	61.23^j^	194.43^defgh^	185.66^efghi^	205.16^abcd^	195.08^ef^
G2	34.47^pq^	43.55^bcd^	35.50^op^	37.84^ghi^	11.09^kl^	14.10^bcdef^	11.73^ijkl^	12.31^hij^	85.12^bcd^	56.47^opq^	92.77^a^	78.12^bcd^	210.68^abcd^	209.33^abcd^	202.60^abcde^	207.54^bc^
G3	36.20^nop^	45.00^b^	36.13^nop^	39.11^de^	11.54^jkl^	16.16^a^	11.66^ijkl^	13.12^efg^	73.86^ghij^	63.44^lmno^	69.61^ijkl^	68.97^gh^	213.79^abcd^	185.37^efghi^	209.70^abcd^	202.95^bcde^
G4	36.50^mon^	44.06^bc^	38.38^hijk^	39.65^de^	11.90^ijkl^	14.96^bc^	12.46^ghij^	13.11^efgh^	71.04^hijkl^	53.04^pqr^	64.42^klmno^	62.83^ij^	206.60^abcd^	194.96^defgh^	207.86^abcd^	203.14^bcde^
G5	40.23^fgh^	40.63^fgh^	41.60^def^	40.82^c^	14.06^bcdef^	13.70^cdefg^	13.53^cdefg^	13.76^cde^	64.03^lmno^	36.36^s^	49.22^r^	49.87 ^l^	186.33^efghi^	196.87^cdefg^	207.33^abcd^	196.84^def^
G6	36.29^no^	34.61^pq^	37.43^klmn^	36.11^k^	11.50^jkl^	12.09^hijk^	11.70^ijkl^	11.76^jk^	76.75^efgh^	67.10^jklm^	67.94^ijklm^	70.60^fgh^	216.03^abc^	186.63^efghi^	218.16^a^	206.94^bcde^
G7	33.18^qr^	39.86^ghij^	39.30^ghij^	37.45^hi^	11.13^jkl^	14.30^bcde^	12.86^efghi^	12.76^ghi^	92.17^a^	61.00^mnop^	90.90^ab^	81.36^b^	197.96^cdefg^	179.45^ghi^	205.45^abcd^	194.29^efg^
G8	38.54^hijk^	36.85^lmno^	41.66^def^	39.02^ef^	13.69^cdefg^	12.25^ghij^	14.10^bcdef^	13.34^defg^	71.59^hijk^	82.87^cdef^	64.36^klmno^	72.94^efg^	181.50^ghi^	200.85^bcdef^	195.83^defgh^	192.73^fgh^
G9	43.10 ^cd^	42.75 ^cd^	42.53^de^	42.79^b^	13.66^cdefg^	14.33^bcd^	14.10^bcdef^	14.03^bcd^	64.38^klmno^	66.52^klm^	74.72^ghij^	68.54^h^	215.60^abc^	198.40^bcdefg^	201.99^abcde^	205.33^bcd^
G10	37.27^lkmn^	38.86^hijk^	38.36^ijkl^	38.16^fgh^	12.47^ghij^	13.43^defgh^	12.26^ghij^	12.72^ghi^	73.47^ghij^	68.07^ijklm^	78.81^defgh^	73.45^ef^	198.77^bcdefg^	189.62^efgh^	212.80^abcd^	200.39^cdef^
G11	33.20^qr^	34.24^pq^	36.33^no^	34.59^l^	11.03^kl^	11.78^ijkl^	11.70^ijkl^	11.50^kl^	83.11^cde^	75.25^efgh^	81.37^cdef^	79.91^bc^	201.03^abcde^	190.66^defgh^	210.52^abcd^	200.74^bcdef^
G12	46.94^a^	42.00^def^	43.26 ^cd^	44.07^a^	15.66^a^	15.00^b^	15.30^b^	15.32^a^	57.86^nopq^	64.53^klmno^	66.09^klmn^	62.83^ij^	170.92^i^	180.03^ghi^	182.80^fghi^	177.92^i^
G13	40.96^ef^	40.00^fgh^	39.20^ghij^	40.05^cd^	14.44^bcd^	14.36^bcd^	14.60^bcd^	14.46^bc^	59.28^nop^	48.05^r^	55.96^opq^	54.43^k^	168.42^j^	178.66^hi^	184.30^fghi^	177.12^i^
G14	34.37^pq^	28.63^s^	37.53^klmn^	33.51^m^	11.39^jkl^	8.96^m^	12.40^ghij^	10.92 ^l^	83.43^bcde^	84.39^bcd^	92.17^a^	86.66^a^	201.43^abcde^	219.32^a^	202.79^abcde^	207.84^b^
G15	39.34^ghij^	38.66^hijk^	39.20^ghij^	39.07^ef^	13.45^defgh^	14.02^bcdef^	13.56^cdefg^	13.67^def^	68.18^ijkl^	64.42^klmno^	67.26^ijklm^	66.62^hi^	194.06^defgh^	175.92^i^	188.92^efgh^	186.30^ghi^
G16	32.39^r^	35.93^nop^	35.53^op^	34.61^l^	10.71^ l^	12.16^hijk^	11.96^ijkl^	11.61^jkl^	85.99^abc^	79.02^defgh^	80.58^cdefg^	81.86^b^	202.18^abcde^	195.43^defgh^	196.38^cdefg^	197.99^cdef^
G17	38.32^ijklm^	37.53^klmn^	40.83^efg^	38.89^ef^	13.31^defgh^	13.56^cdefg^	14.16^bcdef^	13.68^def^	73.54^ghij^	77.02^efgh^	75.02^fghi^	75.19^de^	188.23^efghi^	177.25^hi^	188.35^efghi^	184.61^hi^
G18	38.06^jklm^	36.36^no^	36.80 ^lmno^	37.07^ij^	12.48^ghij^	11.83^ijkl^	11.60^ijkl^	11.97^ijk^	80.33^cdefg^	63.72^lmno^	85.05^bcd^	76.37^cde^	205.10^abcd^	207.32^abcd^	217.20^ab^	209.87^a^
G19	36.74^lmno^	35.03^op^	37.50^klmn^	36.42^jk^	11.93^ijkl^	11.90^ijkl^	12.30^ghij^	12.04^ijk^	86.87^abc^	72.51^ghijk^	75.88^efgh^	78.42^bcd^	208.43^abcd^	195.35^defgh^	205.07^abcd^	202.95^bcde^
G20	41.60^def^	46.53^a^	44.26^bc^	44.13^a^	13.45^defgh^	16.76^a^	14.03^bcdef^	14.75^ab^	72.33^hijk^	75.58^efgh^	71.67^hijk^	73.19^ef^	209.43^abcd^	179.05^ghi^	215.43^abc^	201.30^bcde^
Mean	37.75^b^	38.99^a^	38.99^a^	38.58	12.58^c^	13.46^a^	12.93^b^	12.99	74.37^a^	65.49^b^	73.65^a^	71.17	194.43^a^	191.31^b^	202.14^a^	197.59

Means followed by the same letter are not significantly different (*P *≤* *0.01) from each other, according to Duncan's multiple range test.

The results showed significant differences (*P *≤* *0.01) in dry gluten (DG) among genotypes, growing environments, and the genotype‐environment interaction (Table 5). The DG values for the genotypes in the three environments ranged from 10.71 to 15.66% at Wad Medani, 8.96 to 16.76% at Hudeiba, 11.60 to 15.3% at Dongola. Concerning growing area, DG content was higher at Hudeiba followed by Dongola and then Wad Medani. Regardless of the growing environment and throughout all genotypes, the highest mean for DG was obtained for G12 while the lowest value was obtained for G14. Regarding the interaction, the highest value was obtained for G20 (16.76%) and G3 (16.16%) at Hudeiba and G12 (15.66%) at Wad Medani, while the lowest value (8.96%) was obtained for G14 in Hudeiba. The yield of DG was closely associated with the total protein of these wheat lines. These results agreed with those reported previously for other of wheat genotypes (Makawi et al. 2013).

The gluten index (GI) is a predictive method of gluten strength and thus it is a good indicator for gluten quality and quantity (Vida et al. 2014). Wide variations (*P *≤* *0.01) in the GI of 20 wheat genotypes grown in three different environments were explicitly noted (Table 5) and associated with genotypes, growing environments, and the interaction between these factors. The range values of GI for the genotypes were 57.86–92.17%, 36.36–84.39%, and 49.22–92.77% of the growing environments Wad Medani, Hudeiba, and Dongola, respectively. Strikingly, the gluten index of all wheat genotypes fall within the optimal range (55–100) for breadmaking (Har Gil et al. 2011; Makawi et al. 2013) when they grow in Wad Medani. Throughout the three growing environments, the highest mean for GI obtained were 92.77% and 92.17% at Dongola and Wad Medani, respectively, while the lowest value (36.36%) was observed at Hudeiba. Among genotypes and regardless of the environment, the results showed that G14 has the highest (86.66%) mean value of GI while G5 has the lowest value. Our findings demonstrated that genotypes, growing environments, and their interaction significantly affected GI, with the highest effect being from the genotypes. In agreement with our findings, Vida et al. (2014) reported that the gluten index had the greatest dependence on the genotype compared to environmental factors and agronomic treatments. Furthermore, the more significant effect of genotype on the gluten index compared to the impact of environment and fertilizer application was recently reported (Bouacha et al. 2014). GI correlated positively (*P <* *0.05) with WHC (*r* = 0.53) at Wad Medani and with SV (*r* = 0.50) at Dongola, while it showed negative correlations with WG at Wad Medani (***P <* *0.001, *r* = −0.83), Hudeiba (*P <* *0.05, *r* = −0.47) and Dongola (*P <* *0.05, *r* = −0.48) (Table 3). GI also correlated negatively with DG at Wad Medani (***P <* *0.001, *r* = −0.86) and Dongola (*P <* *0.05, *r* = −0.51), and with PC at Hudeiba (**P <* *0.01, *r* = −0.61). These results indicate a contradicting response between GI and the three major wheat quality parameters (PC, WG, and DG) and therefore much concern has to be considered when using GI for wheat quality evaluation (Bonfil and Posner 2012; Kaur et al. 2013).

The results showed significant differences (*P *≤* *0.01) in the water holding capacity (WHC) among environments and genotypes as well as the interaction between genotypes and environments (Table 5). The mean values of WHC were 168.42–216.03%, 175.92–219.32%, and 182.8–218.16% for the genotypes grown at Wad Medani, Hudeiba, and Dongola, respectively. Throughout the three growing environments, WHC showed extremely negative (***P <* *0.001) correlation with DG (*r* = −0.64 to −0.70), whereas the correlations between WG and DG were highly positive (***P <* *0.001, *r* = 0.87–0.96) (Table 3). The highest mean percentages for WHC of gluten was obtained for G14 (219.32%) and G6 (218.16%) grown at Hudeiba and Dongola, respectively, while the lowest value was observed for G13 (168.42%) cultivated at Wad Medani. Throughout all the three environments, the results indicated highest WHC for G18 while the lowest value was obtained for G13. Between environments, not all varieties varied in the same manner; however, some had the same general score in all areas, whereas others varied. This variation may be due to the effect of environmental conditions such as heat stress, soil conditions, and agronomical practices.

### Falling number

Statistical analysis revealed significant differences (*P *≤* *0.01) in the mean falling number (FN) of 20 wheat genotypes grown in three different environments (Table 6), indicating that environmental conditions had influenced the flour FN. In addition, the interaction between genotypes and growing environments was also significantly affected the *α*‐amylase activity. The FN values were ranged from 532.67 to 715.0 sec, 508.00 to 656.67 sec, and 594.33 to 974.67 sec for the genotypes cultivated at Wad Medani, Hudeiba, and Dongola, respectively. These results were in good agreement with the data reported by Kaur et al. (2013) who found that the falling number of Indian wheat cultivars was high and ranged from 485 to 967 sec. Through the three environments, the highest FN was obtained at Dongola while the lowest value was obtained at Hudeiba. FN correlated positively with SV (*P <* *0.05, *r* = 0.47), WG (*P <* *0.05, *r* = 0.48), and FC (***P <* *0.001, *r* = 0.67) at Hudeiba, while it showed negative correlation with DG (*P <* *0.05, *r* = −0.45) at Wad Medani (Table 3). Among all genotypes, the highest falling number recorded for G2 while the lowest value was obtained for G5. The Sudanese wheat genotypes possess very high FN and thus indicate flours with a little *α*‐amylase activity. This could be attributed to the dry weather during grain filling and harvesting time, which consequently affect the activity of *α*‐amylase (Erekul and Kohn 2006; Hamad et al. 2013). Thus, the seasonality and the environment, storage conditions of wheat grain (moisture and temperature), had a significant impact on the *α*‐amylase activity. Previous reports indicate that the FN is diverse among different genotypes that cultivated in various environment (Hamad et al. 2013) with the environmental impact on the FN being higher than the genotype and the genotype‐environment interaction (Erekul and Kohn 2006).

**Table 6:** Falling number and sedimentation value of 20 local wheat genotypes grown in three environments

Genotypes	Falling number (sec)				Sedimentation value (mL)			
	Wad Medani	Hudeiba	Dongola	Mean	Wad Medani	Hudeiba	Dongola	Mean
G1	609.67^r^	508.00^z^	650.33^n^	589.33^k^	23.00^k^	22.00^l^	23.33^k^	22.78^l^
G2	633.33^p^	636.33^op^	974.67^a^	748.11^a^	25.33^i^	30.00^d^	24.00^j^	26.44^f^
G3	693.00^gh^	556.33^wx^	648.67^n^	632.67^h^	27.00^g^	29.00^e^	24.00^j^	26.67^e^
G4	715.00^f^	656.67^mn^	665.00^kl^	678.89^c^	24.00^j^	27.00^g^	26.00^h^	25.67^g^
G5	563.33^w^	524.00^z^	600.00^rs^	562.44^m^	23.00^k^	24.00^j^	24.00^j^	23.67^k^
G6	672.33^jk^	528.33^z^	808.67^b^	669.78^d^	19.00^n^	22.00^l^	24.00^j^	21.67^m^
G7	673.33^jk^	540.33^y^	730.33^e^	648.00^f^	25.00^i^	24.00^j^	40.33^a^	29.78^b^
G8	685.33^hi^	595.33^st^	795.00^c^	691.89^b^	25.00^i^	32.00^b^	27.00^g^	28.00^c^
G9	589.00^tu^	556.67^wx^	668.67^kl^	604.78^j^	32.00^b^	28.33^f^	33.00^b^	31.11^a^
G10	594.67^st^	508.33^z^	604.33^rs^	569.11^l^	28.00^f^	26.00^h^	29.00^e^	27.67^d^
G11	687.33^hi^	547.00^xy^	733.00^e^	655.78^e^	23.00^k^	24.00^j^	24.33^j^	23.78^k^
G12	604.00^rs^	555.67^wx^	653.33^mn^	604.33^j^	27.00^g^	24.00^j^	24.00^j^	25.00^i^
G13	607.00^r^	623.67^q^	654.67^mn^	628.44^h^	23.00^k^	23.00^k^	23.00^k^	23.00^l^
G14	668.33^kl^	512.00^z^	759.67^d^	646.67^f^	19.00^n^	23.00^k^	28.00^f^	23.33^l^
G15	636.00^op^	576.67^v^	681.67^i^	631.44^h^	25.00^i^	23.00^k^	25.00^i^	24.33^j^
G16	583.67^uv^	577.00^v^	701.33^g^	620.67^i^	20.00^m^	27.00^g^	29.00^e^	25.33^h^
G17	532.67^z^	583.00^uv^	594.33^st^	570.00^l^	28.00^f^	26.00^h^	29.33^e^	27.78^d^
G18	681.33^ij^	580.33^uv^	600.33^rs^	620.67^i^	27.00^g^	27.00^g^	29.00^e^	27.67^d^
G19	638.33^op^	596.67^st^	662.67^lm^	632.56^h^	26.00^h^	23.00^k^	28.67^ef^	25.89^g^
G20	626.67^pq^	646.00^no^	648.00^n^	640.22^g^	31.00^c^	28.00^f^	30.33^cd^	29.78^b^
Mean	634.72^b^	570.42^c^	691.73^a^	632.29	25.02^c^	25.62^b^	27.27^a^	25.97

Means followed by the same letter are not significantly different (*P *≤* *0.01) from each other, according to Duncan's multiple range test.

### Sedimentation values

The sedimentation value (SV) assessment provides information on the protein quantity and the quality of wheat flour (Makawi et al. 2013). It is thus used as a screening tool in wheat breeding programs as well as in milling and breadmaking processes. Our results revealed significant differences (*P *≤* *0.01) in SV among environments and genotypes (Table 6). The SV of the genotypes in the three environments was in the range of 19.00–32.00 mL at Wad Medani, 22.00–32.00 mL at Hudeiba and 23.00–40.33 mL at Dongola. Similarly, Makawi et al. (2013) stated that the sedimentation value of the three Sudanese cultivars (Debaira, WadiElneel, and Elneelain) ranged between 19.6 and 37.4 mL. Additionally, Kaya and Akcura (2014) found the sedimentation value of 24–33 mL for Turkish wheat genotypes grown in different environments. The highest mean SV (40.33 mL) was obtained from G7 grown at Dongola while the lowest value (19.00 mL) was obtained from G6 and G14 grown at Wad Medani. Throughout the three environments, genotypes grown at Dongola showed the highest SV followed by Hudeiba and then Wad Medani. SV on the other hand revealed positive correlation with WG at Wad Medani (*P <* *0.05, *r* = 0.55) and Hudeiba (*P <* *0.05, *r* = 0.45), and with PC (***P <* *0.001, *r* = 0.69) at Wad Medani, and FC (**P <* *0.01, *r* = 0.59) at Hudeiba (Table 3). The positive association of SV with PC and WG is consistent with the fact that this value depends mainly on the wheat protein composition and gluten quality and is frequently correlate with these quality attributes (Ozturk and Aydin 2004; Tahir et al. 2006; Kaya and Akcura 2014). Unrelatedly with the growing area, G9 expressed the highest mean SV. As for other traits, the SV of the current study also depend mainly on the genotype, the growing environment (temperature and soil fertility) and their interaction. Similar observation on the effect of environment (temperature, rainfall, and soil quality) and agronomical treatments on the sedimentation value of many wheat genotypes has been previously reported (Erekul and Kohn 2006; Tahir et al. 2006; Kaya and Akcura 2014).

### Biplot analysis

**Figure 1:**
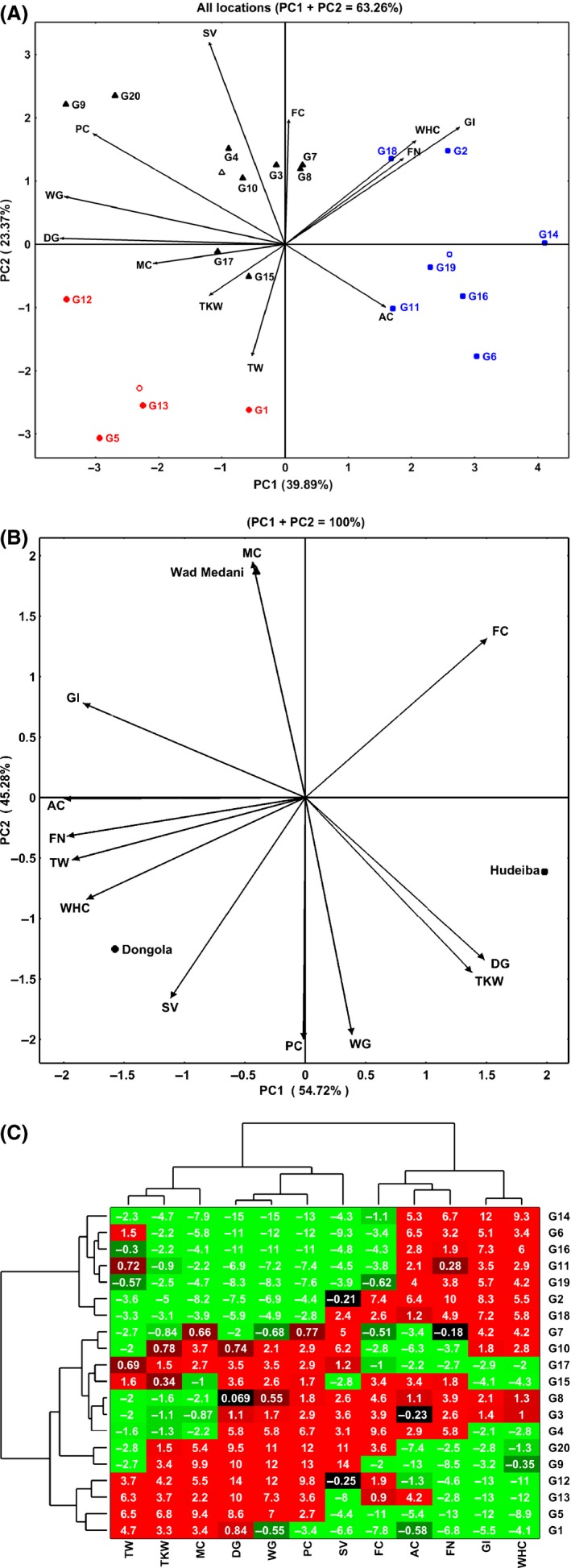
Biplot based on principal component analysis for grain quality traits in 20 wheat genotypes (G1–G20) grown in three different environments (Wad Medani, Hudeiba, and Dongola). The biplots showed the interrelations between the quality traits (A) and the environments (B). Bidimensional clustering analysis is presenting the relationships between the genotypes (C). TKW, Thousand kernel weight (g); TW, Test weight (kg/hL); AC, Ash content; FC, Fat content; PC, Protein content; FN, Falling number; WHC, Water holding capacity; GI, Gluten index; SV, Sedimentation value; WG, Wet gluten; DG, Dry gluten.

To profoundly determine the multivariate relationships between the grain end‐use quality traits and the growing environments of 20 wheat genotypes, biplot analysis was carried out by comparing the eigenvalues of PC1 and PC2 of principal component analysis (PCA) for both the genotypes and the quality traits (Fig. 1A–C). Regarding the interrelation between the traits and genotypes, the results of the first two PC axes (PC1, 39.89% and PC2, 23.37%) accounted for about 63.26% of the total variability reflecting the complexity of the variation between the plotted components (Fig. 1A). In the biplot, vectors of traits (variables) showing acute angle are positively correlated, whereas those formed obtuse or straight angles are negatively correlated, and those with right angle have no correlation. The distance between the raw (genotypes) is interpreted in terms of similarity. Regarding the traits, PC1 had the breadmaking quality parameters (DG, WG, PC, GI, and WHC) as the principal components, and FN and MC to a lesser extent while, PC2 had the SV, FC, and TW as the primary elements. The cosine of the angles between vectors indicated a high positive correlation between WHC, FN, and GI in the positive direction. These three traits were also positively correlated with FC in the positive direction and AC in the negative direction. High positive correlation was also observed between PC, WG, and DG and between SV and FC, and similarly between TW, TKW, and MC. In contrast, WHC, FN, and GI were negatively correlated with other breadmaking quality parameters mainly PC, WG, and DG and with grain physical characteristics such as TW, TKW, and MC. The SV was also negatively correlated with AC, TW, and TKW. Overall, the biplot analysis exhibits three groups of the traits based on their phenotypic associations, those include; gluten, starch, and milling quality characteristics (GI, WHC, FN, and AC) group, breadmaking quality attributes (SV, PC, WG, and DG) group, and grain physical and marketing characteristics (MC, TKW, and TW). These results shows some differences from that of the correlation analysis among pairs of characters as the biplot describes the interrelationships among all characters concurrently based on the overall contribution of the data (Yan and Fregeau‐Reid 2008).

Additionally, the biplot could indicate the interrelation among the genotypes as well as their stability and contribution toward an individual trait (Morris et al. 2004). In this regards, hierarchical clustering clearly distinguished three groups of genotypes according to their quality characteristics in all growing environments. The first group (right half of the graph, square symbol) is formed by the genotypes (G2, G8, G11, G14, G16, G18, and G19) with the highest values of WHC, GI, FN, and AC compared to other genotypes. Within this group, G14 had the highest values for these traits, followed by G6, G16, G12, G11, and G18. The second group (upper left, triangle symbol) consists of G3, G4, G7, G8, G9, G10, G15, G17, and G20. This group characterized by its high breadmaking quality parameters such as PC, WG, DG, and SV with G9 and G20 outscore all others genotypes for these traits. However, G9 and G20 are less stable for these quality traits compared to the other genotypes in this group as well as they are not contributed to the other quality traits such as FN, GI, and WHC. By contrast, G3, G4, G7, G8, and G10 are more stable and well‐associated with all end‐use quality attributes. The last group (lower left, circle symbol) contain G1, G5, G12, and G13, those characterized mainly by their high values of grain milling and marketing characteristics especially TW, TKW, and MC. Like that of traits, the results of biplot analysis display three distinguished groups of the genotypes based on their performance for one or more quality traits. Saint Pierre et al. (2008) stated that the grouping of genotypes in the biplot indicated that the genotypes of the quality groups show similar performance to numbers of the quality traits.

The biplot is an appropriate method for the analysis of the interaction between the traits and environments. Thus, it can identify the effects of the environment on one or more characters through a range of genotypes. In this study, a biplot was formed by using the average means of each trait for all genotypes growing in three environments to find the better wheat‐growing environment for the end‐use quality attributes (Fig. 1B). The results showed extremely high variability (100%), arising from the first two principal component PC1 (54.92%) and PC2 (45.28%), which indicating an excellent contribution of these two axes to the data presentation. Interestingly, the association between the traits shows some variations especially in MC, FC, TW, and FN compared to those presented in Figure 1A, suggesting the effect of the growing environment on grain end‐use quality traits. The high MC and GI characterize the genotypes grown at Wad Medani compared to the same genotypes when cultivated in the other two environments. Higher MC at Wad Medani could be attributed to the soil type in this environment which it could retain more water than that of the two other environments. The genotypes grown at Hudeiba had higher DG, TKW, and FC compared to the same genotypes grown at Wad Medani and Dongola. Interestingly, most of the end‐use quality parameters are great in the genotypes when cultivated at Dongola compared to the other two growing environments. Based on the end‐use quality traits, the environment in Dongola is most suitable followed by that of Hudeiba, whereas, the environment in Wad Medani is not suitable for wheat cultivation as the end‐use quality attributes were significantly reduced in this environment. The chief difference between the three environments is the temperature. Therefore, the inferior quality of the genotypes at Wad Medani could be attributed to the high temperature during the growing season.

To select the best genotype based on its quality performance throughout the growing environments, we generated a bidimensional cluster from the mean of the quality attributes of each genotype across all environments (Fig. 1C). The horizontal axis groups the genotypes based on phenotypic similarity concerning their quality traits. The differences in the color intensity indicated the values of each feature with the red color being the highest and green is the lowest. The two major branches of the horizontal cluster (traits) discrete the genotypes in the upper clusters, in which most of the green color (small values) appears for the attributes TW, TKW, MC, DG, WG, PC, and SV, from those in the lower clusters as they showed red color (high values) for the same attributes. With view exceptions, this results suggests that the upper branch includes genotypes (G14, G6, G16, G11, and G19) with poor grain filling and end‐use quality, whereas, the lower branch contains G9, G20, G10, G15, and G17 with real grain weight and end‐use quality. Despite their poor grain filling and moisture content, G3 and G4 have a good end‐use quality attributes with G3 outscore all other genotypes in this regard. Strikingly, this genotype (G3) also shows good stability for these end‐use quality traits (Fig. 1A). These results indicate the potentiality of G3 as an excellent and stable genotype for end‐use quality attributes under hot environments.

## Conclusion

In conclusion, the results of this study demonstrate that the genotypes, the environment, and their interaction have a high impact on the end‐use quality attributes of Sudanese wheat genotypes grown in three different environments. Throughout the three growing environments, Dongola is most appropriate for producing wheat grains with adequate end‐use quality characteristics while Wad Medani is the least in this regard due to its high temperature. Among wheat genotypes, G3 and G4 exhibit good performance and reasonable stability for most of the tested quality traits. These genotypes are potentially excellent candidates for cultivation in the hot environments of the Sudan for producing wheat grains with good breadmaking quality. In addition, the crossing of these genotypes with high yield and milling quality genotypes will improve the adaptability, productivity, quality, and marketability of Sudanese wheat grains.

## Conflict of Interest

The authors declare to have no conflict of interest.
